# Hto, Tritiated Amino Acid Exposure and External Exposure Induce Differential Effects on Hematopoiesis and Iron Metabolism

**DOI:** 10.1038/s41598-019-56453-4

**Published:** 2019-12-27

**Authors:** Jean-Marc Bertho, Dimitri Kereselidze, Line Manens, Cécile Culeux, Victor Magneron, Joel Surette, Melinda Blimkie, Linsdey Bertrand, Heather Wyatt, Maâmar Souidi, Isabelle Dublineau, Nicholas Priest, Jean-René Jourdain

**Affiliations:** 1 Institut de Radioprotection et de sûreté nucléaire (IRSN), PSE-SAN/SESANE, LRTOX, Fontenay-aux-Roses, France; 2 Canadian Nuclear Laboratories Ltd., Radiological Protection Research and Instrumentation Branch, Chalk River, Ontario, Canada; 3 Institut de Radioprotection et de sûreté nucléaire (IRSN), PSE-ENV, SEDRE, USDR, Fontenay-aux-Roses, France; 4 Institut de Radioprotection et de sûreté nucléaire (IRSN), DAI, Fontenay-aux-Roses, France

**Keywords:** Risk factors, Public health

## Abstract

The increased potential for tritium releases from either nuclear reactors or from new facilities raises questions about the appropriateness of the current ICRP and WHO recommendations for tritium exposures to human populations. To study the potential toxicity of tritium as a function of dose, including at a regulatory level, mice were chronically exposed to tritium in drinking water at one of three concentrations, 10 kBq.l^−1^, 1 MBq.l^−1^ or 20 MBq.l^−1^. Tritium was administered as either HTO or as tritiated non-essential amino acids (TAA). After one month’s exposure, a dose-dependent decrease in red blood cells (RBC) and iron deprivation was seen in all TAA exposed groups, but not in the HTO exposed groups. After eight months of exposure this RBC decrease was compensated by an increase in mean globular volume - suggesting the occurrence of an iron deficit-associated anemia. The analysis of hematopoiesis, of red blood cell retention in the spleen and of iron metabolism in the liver, the kidneys and the intestine suggested that the iron deficit was due to a decrease in iron absorption from the intestine. In contrast, mice exposed to external gamma irradiation at equivalent dose rates did not show any change in red blood cell numbers, white blood cell numbers or in the plasma iron concentration. These results showed that health effects only appeared following chronic exposure to concentrations of tritium above regulatory levels and the effects seen were dependent upon the speciation of tritium.

## Introduction

Tritium is the main radionuclide released by current nuclear power plants (NPP) at an estimated level of 0.1 EBq per year^1^ - and future nuclear fission reactors (such as the ITER reactor) will increase these releases. Environmental and human health effects, especially through water contamination, have the potential to be caused by these releases. Current tritium regulations are most commonly based on the recommendations of the International Commission on Radiological Protection (ICRP) and of the World Health Organization (WHO) which recommend limits on the annual exposure from radionuclides in drinking water to 0.1 mSv^2^. This can be compared to the estimated annual 0.01 µSv from tritium of natural origin^1^. Using the ICRP biokinetic model for tritium internal exposure, and assuming tritium to be the sole contributor to the dose, the annual effective dose of 0.1 mSv corresponds to a level of tritium in drinking water of about 7.6 kBq.l^−1^ ^3^. As a result, WHO recommends a regulatory limit of 10 kBq.l^−1^ for the tritium content in drinking water^2^. Nevertheless, national standards for tritium in drinking water vary by several orders of magnitude, from 100 Bq.l^−1^ in the European Union up to 76 kBq.l^−1^ in Australia, with most of countries adopting a regulatory level close to or below 10 kBq.l^−1^, in accordance with the WHO recommendation^2,4^. Discrepancies between national regulations reflect the different applications of the ICRP radiation protection principal that requires radiation exposures to be as low as reasonably achievable (ALARA). They also reflect uncertainties in our understanding of the potential toxicity of tritium^5^.

Predicting the consequences of intakes of tritium are problematic. The first problem is the low energy (mean 5.7 keV, max 18 keV) of the beta rays emitted as tritium disintegrates, which results in a short energy deposition track (<6 µm in water)^6^. Consequently, tritium poses a health risk only if internalized. The second problem results from the many chemical forms in which tritium can be found. The major chemical form of tritium in the environment is tritiated water (HTO)^7^, but there are other forms where tritium has been incorporated into compounds with a covalent link to a carbon atom – referred to as organically-bound tritium (OBT). OBT accounts for approximately 5–30% of tritium in living organisms^8^, even in humans^9,10^. OBT forms include sugars, proteins, lipids and other organic compounds. It follows, the biokinetics of tritium depends on its chemical speciation. The HTO form is considered to be of low toxicity, due to its homogeneous distribution and the rapid turn-over of water in the body. In contrast, some OBT forms have very different biokinetics with residence times in organs and cells which are much longer than for HTO^3^. OBT forms are able to be incorporated within the cell and its nucleus, resulting in a higher probability of lethal damage to the cell^11–13^. It is therefore speculated that risks associated with tritium internal exposures may be underestimated^14,15^.

Epidemiological studies exploring the health effects of tritium incorporation are few and provide little information on tritium toxicity^1,16^. In part, this is because occupational tritium doses are generally integrated into total occupational dose, and thus it is not possible to analyze the risks associated with tritium alone^17^. In some cases, an analysis was conducted taking account of tritium exposure separately, but these studies were not informative because of insufficient statistical power^18,19^. It follows that most studies of the health effects of the internal exposure to tritium have used animal models. These studies have shown teratogenic^20^, carcinogenic^21^ and hematopoiesis-linked lethality^22^. However, most of these studies employed acute exposure to tritium quantities in the GBq range. Such exposures are several orders of magnitude higher than environmental concentrations - even in the vicinity of nuclear plants^23,24^ and in most of the cases exposures are to HTO^1^. In contrast, studies of the health effects produced by chronic exposure to environmentally relevant concentrations of tritium are rare. Some chronic exposure studies showed a shortening of lifespan, due to carcinogenicity, but they employed tritium concentrations corresponding to 4–250 mGy per day^21,25^, so again several orders of magnitude higher than current regulatory levels. To fill the knowledge gap, a large-scale *in vivo* mouse study was conducted to study the biokinetics and non-cancerous/carcinogenic effects of tritium incorporation at low concentrations relevant to possible human exposures and current regulations. Details about this large-scale study have been published^26^. Two different forms of tritium were used, HTO and OBT (in the form of a mixture of three amino acids (AA), alanine, glycine and proline). These amino acids (hereafter referred to as TAA) were chosen for several reasons: They are highly soluble in water so the exposure is easy to manage by their addition to drinking water^27,28^; They are non-essential amino acids that participate in normal AA metabolic processes; They are frequent in vertebrate proteins^29^; With the exception of proline, they are non-functional AA, i.e. not implicated into key metabolic pathways^30^. Proline is implicated in several metabolic pathways, including osmotic regulation; stress protection; cellular signaling processes and more recently discovered role in cancer cell metabolism^30^.

Finally, because of the short track range (<6 µm) and the low energy of tritium beta rays^6^ tritium is considered a low radiotoxicity radionuclide. However, at the micro-dosimetric level concentrated energy deposition can occur – particularly following the incorporation of OBT into structural components of cells and tissues. Such concentrations could result in higher than expected risks^14,15^. To study this, more evenly distributed, gamma-rays from cobalt-60 were employed at the same doses and dose rates for comparison. In this way the observed biological effects produced by HTO/OBT and gamma rays can be compared. During this study, a decrease in red blood cell number was evidenced after one month’s exposure to TAA, but not to HTO or to an external gamma irradiation at a dose rate equivalent to the one resulting from the internal exposure to tritium. In the present work, a detailed analysis of hematologic parameters is presented and the link between observed tritium-induced changes and modifications in iron metabolism are investigated.

## Results

### Blood cell numeration and formula

**Figure 1:**
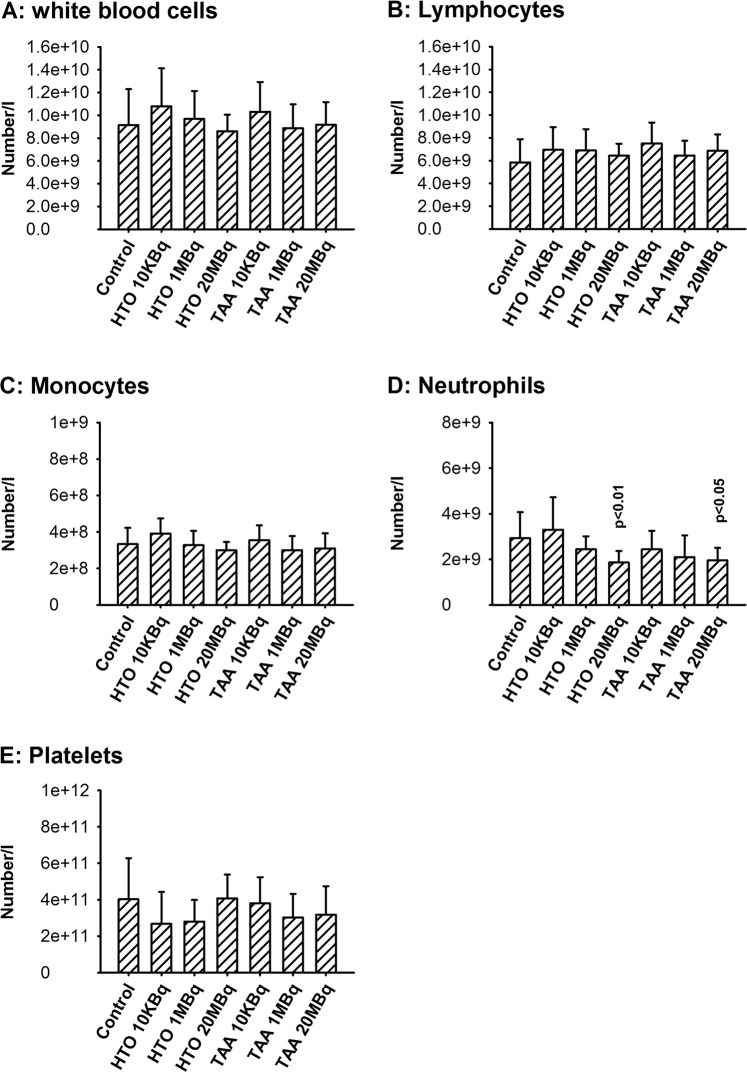
Blood cell counts and differentials in animals after one month of exposure to either HTO or TAA. (**A**) White blood cells; (**B**) lymphocytes; (**C**) monocytes; (**D**) neutrophils; (**E)** platelets. Results are mean ± SD, n = 11. No significant differences were observed in the different exposure groups using a one-way ANOVA test except for neutrophils (H_(6)_ = 16.5, p = 0.011), for which significant differences with the control group are seen.

**Figure 2:**
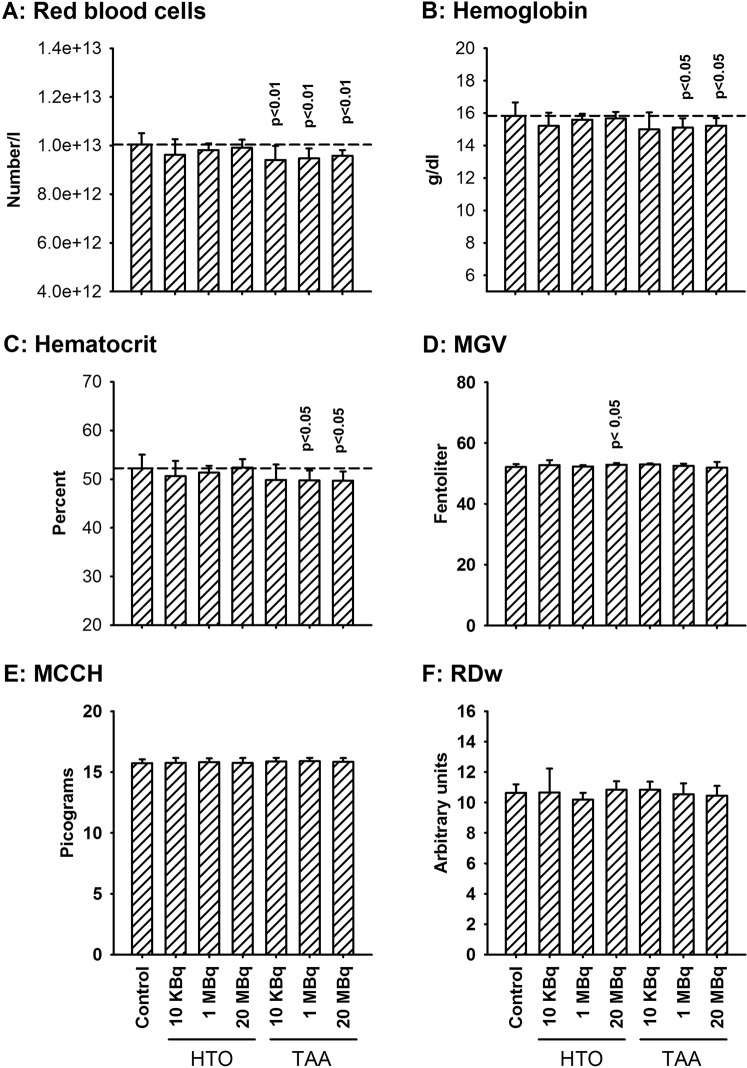
Red blood cell parameters in animals after one month of exposure to either HTO or TAA. (**A**) Red blood cell counts; (**B**) hemoglobin; (**C**) hematocrit; (**D)** mean globular volume (MGV); (**E**) mean corpuscular content in hemoglobin (MCCH); (**F**) RBC distribution index (RDw). Results are mean ± SD, n = 11. Using a one-way ANOVA test significant differences were observed for RBC counts (H_(6)_ = 15.2, p = 0.005), hemoglobin (H_(6)_ = 15.2, p = 0.018), hematocrit (H_(6)_ = 15.4, p = 0.018) and MGV (H_(6)_ = 16.5, p = 0.011) but not for MCCH. Where present, the horizontal dashed line represents the mean value for the control group. The results indicate the appearance of an anemia after one-month’s exposure to TAA but not to HTO.

After one month of exposure to either HTO or TAA, the number and proportion of white blood cells and platelets was mostly unchanged compared to control animals. The exception was a slight decrease in neutrophil numbers in both groups exposed to 20 MBq.l^−1^ of tritium (Fig. 1). In contrast, a significant decrease in the number of red blood cells (RBC) was observed in all TAA-exposed groups, irrespective of tritium concentration, but not in HTO-exposed animals (Fig. 2A). This 4% to 6% decrease in RBC number compared to the control values was associated with decreased hemoglobin concentration, decreased hematocrit and decreased mean corpuscular content in hemoglobin in the 1 and 20 MBq.l^−1^ exposure groups (Fig. 2B–D). These changes were not observed in the HTO groups, except for a slight increase in MGV in the 20 MBq.l^−1^ exposure group. This result indicates the presence of a dose-dependent decrease in RBC in animals exposed to TAA but not in those exposed to HTO. Whether this decrease in RBC parameters amounts might be qualified of anemia is difficult to decide. This is because while the normal median number of RBC in the C57 BL/6J at the age of 3 months is 8.97 × 10^12^.l^−1^ there is a very large range (2.9–11 × 10^12^.l^−1^). Also, there are large variations according to the age, the origin of the mouse strain and the local conditions of animal care^31^. Moreover, there is no defining standard value of RBC or hemoglobin decrease for anemia in the mouse model. In humans, an anemia is defined by a hemoglobin concentration below 130 g.l^−1^ for males. In our hands, the hemoglobin concentration decreased by 4 to 5.2% compared to the control and the RBC numbers by 4–6%. Since this does not represent a major decrease, we choose to use the term “mild anemia”.

**Figure 3:**
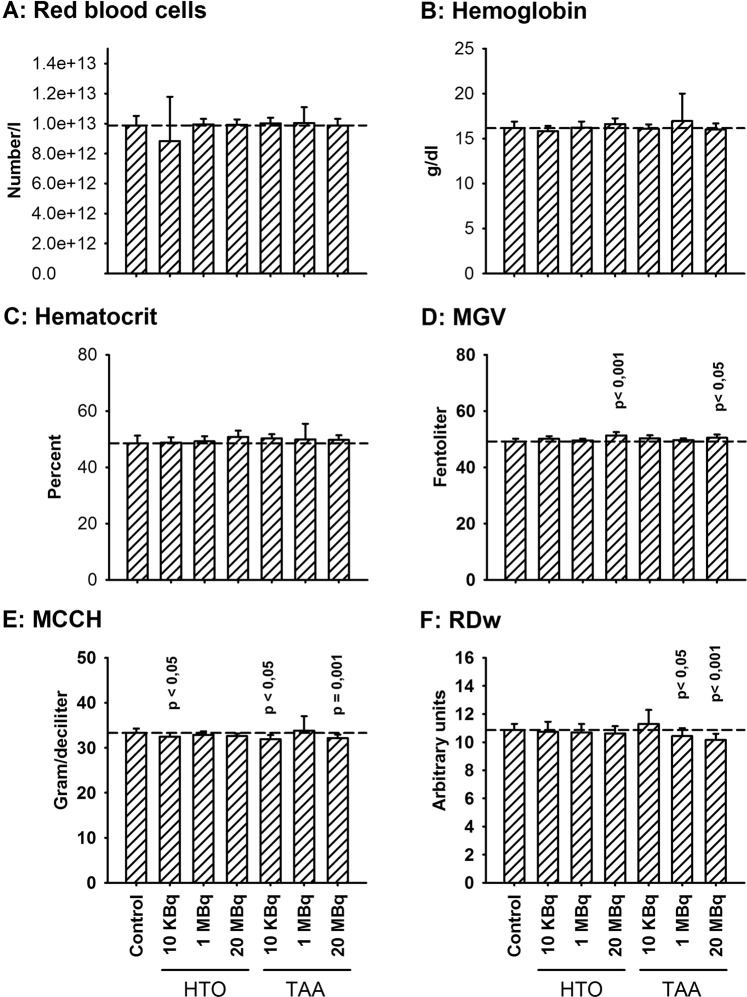
Red blood cell parameters in animals after eight months of exposure to HTO or TAA. (**A**) Red blood cell counts; (**B**) hemoglobin; (**C**) hematocrit; (**D**) mean globular volume (MGV); (**E**) mean corpuscular content in hemoglobin (MCCH); (**F**) RBC distribution index (RDw). Results are mean ± SD, n = 11. Using one-way ANOVA test significant differences were observed for MGV (H_(6)_ = 15.2, p = 0.005), MCCH (H_(6)_ = 15.2, p = 0.018) and RDw (H_(6)_ = 15.4, p = 0.018) but not anymore for RBC counts, hematocrit and hemoglobin. Significant differences with the control group appeared mainly in TAA exposed animals. The horizontal dashed line indicates the mean value for the control group.

After 8 months of exposure, RBC counts returned to control values, as did hemoglobin concentrations and hematocrit (Fig. 3A–C). However, corpuscular parameters showed an increase in the mean RBC volume (MGV) (Fig. 3D), associated with a decrease in mean corpuscular concentration in hemoglobin (MCCH) (Fig. 3E) and a decrease in RBC distribution width (RDw) (Fig. 3F), mainly in animals exposed to TAA at the highest concentration. In HTO-exposed groups, only a slight increase in MGV was observed, but without concomitant changes in other corpuscular parameters. This suggests that the decrease in RBC observed after one month of exposure in TAA exposed animals was compensated with an increased half-time of red blood cells in circulation, leading to increased RBC mean volume and decreased MCCH. This corresponds to a mild anemia induced by TAA exposure, which was not observed in animals exposed to HTO at the same concentration.

### Regulation of RBC differentiation and elimination

An anemia may have several origins, including a defect in erythroid differentiation in the bone marrow, or a defect in RBC retention within the spleen. These two hypotheses were therefore explored.

**Figure 4:**
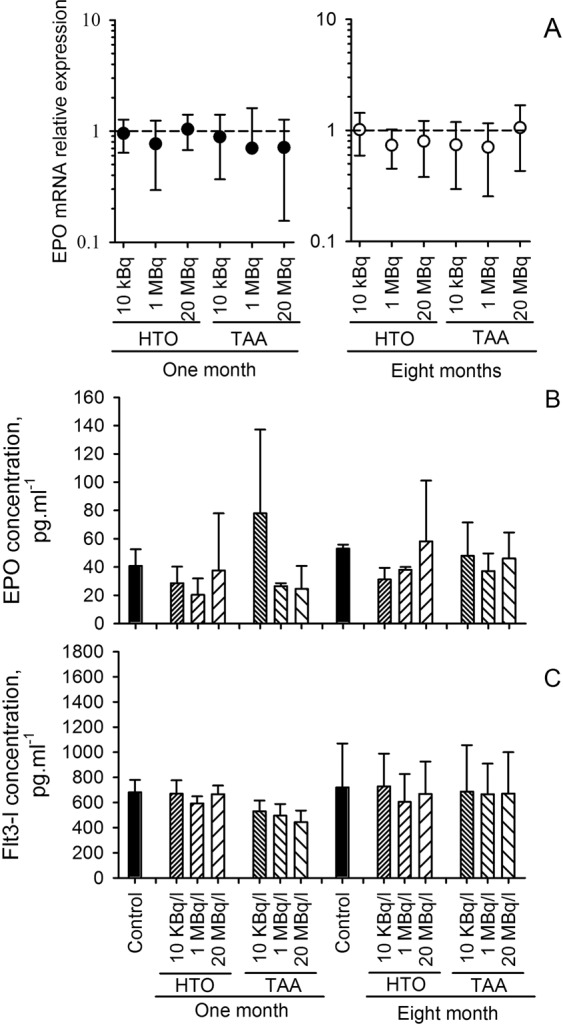
(**A**) Erythropoietin (EPO) mRNA relative expression in the kidney of animals after either one or eight months of exposure to tritium. No significant change in mRNA relative expression was observed cf. the control group (dashed line) (one-way ANOVA test). (**B**) EPO concentration in the plasma. No significant change was observed according to either the group of exposure or the duration of exposure (two-way ANOVA). (**C**) Fms-like tyrosine kinase 3 ligand (Flt3-l) plasma concentration. No significant change was observed as a function of the group of exposure, although a significant increase in Flt3-l concentration was observed with the duration of exposure (two-way ANOVA, F_(1, 132)_ = 7.21, p = 0.008). All results are presented as mean ± SD, n = 11.

Erythroid differentiation from hematopoietic stem cells is strictly regulated by several mechanisms of which cytokine control is of particular importance^32^. We first looked at EPO mRNA expression in the kidney, the main site of EPO production in the body^33^. Results (Fig. 4A) showed the same relative level of EPO mRNA expression in all exposure groups, both after one month and 8 months of exposure, strongly suggesting that EPO production in the kidney was not implicated in the observed mild anemia. We then measured 10 cytokines implicated in the regulation of hematopoiesis in the plasma of animals, and especially EPO which regulates the terminal differentiation of erythroid progenitors^33^ and Flt3-l used as a bio-indicator of hematopoiesis^34,35^. As shown in Fig. 4B,C, no significant differences were observed in the plasma concentrations of Flt3-l and EPO compared to the exposure group. A normal Flt3-l concentration is a strong indicator of normal hematopoietic stem cell differentiation, and a normal EPO concentration is indicative of a normal terminal erythroid differentiation. These results suggest that the origin of the observed anemia at one month was not linked to a deficit in erythropoiesis differentiation. However, one should note that a significant increase in Flt3-l concentration was observed after 8 months of exposure as compared to one month of exposure (two-way anova test, F_(1, 132)_ = 7.21, p = 0.008), especially for the TAA 20 MBq.l^−1^ group (p < 0.05). Since this increase in Flt3-ligand concentration was observed for all experimental groups including the control group, this effect is probably due to the increased age of animals rather than exposure to tritium. Other tested cytokines included G-CSF, M-CSF, SDF-1 and TPO did not show changes compared either to the exposure group or to the duration of exposure (data not shown). The analysis of circulating cytokines confirmed that the decrease in RBC does not result in a defect in hematopoiesis.

**Figure 5:**
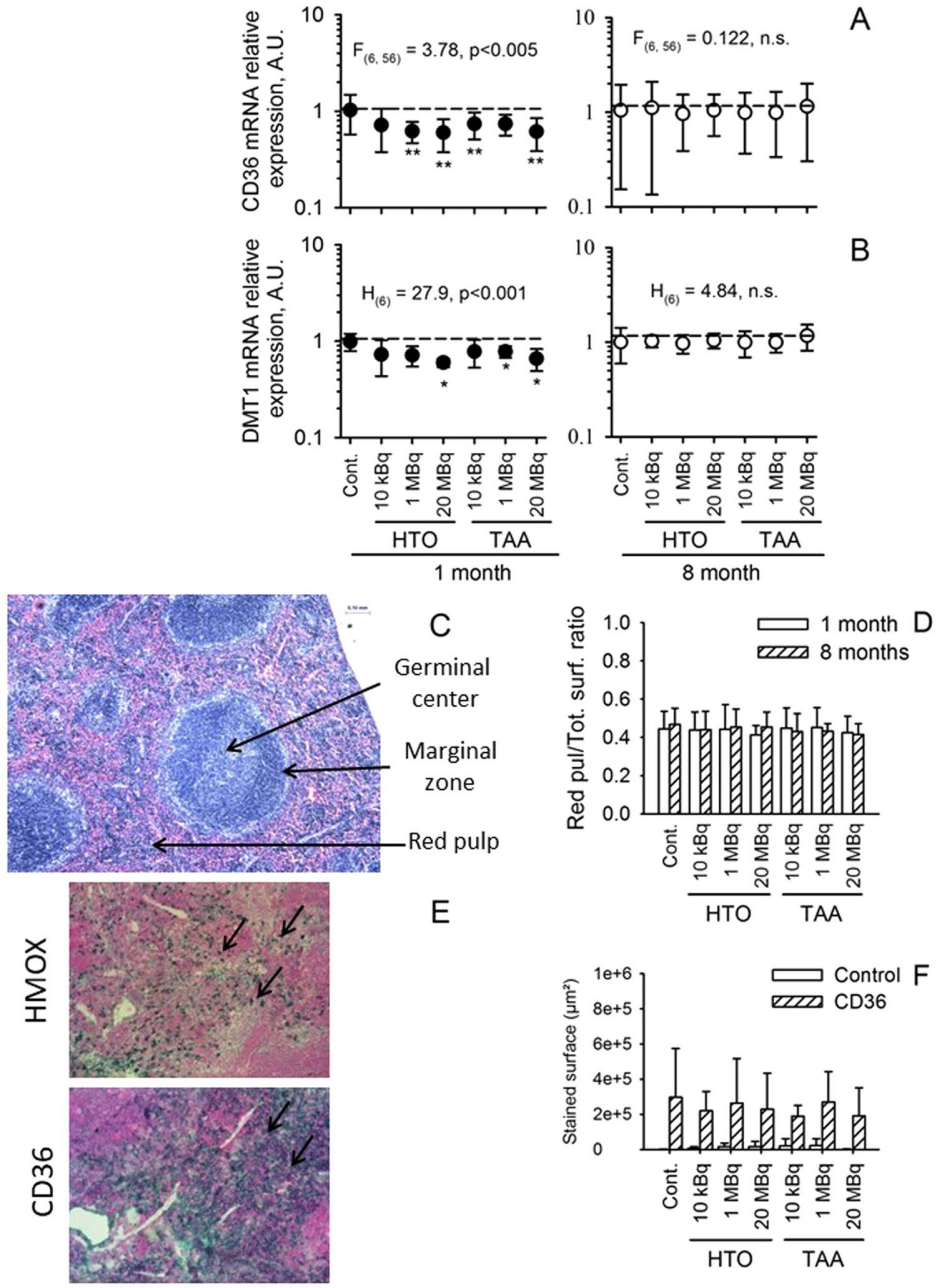
Analysis of the spleen in exposed animals. (**A**) CD36 mRNA and (**B**) DMT1 mRNA relative expression in the spleen of animals after either one or eight months of exposure to tritium. Significant changes in mRNA relative expression was observed after one month of exposure as compared to the control group (dashed line) (one-way ANOVA test), but not after 8 months of exposure. (**C**,**D**) Gross morphological analysis of spleen after one or eight months of exposure. (**C**) Representative morphology of HES-stained spleen in a control animal after one month of exposure, with the red pulp appearing in pink, germinal center as the dark blue area and marginal zone appearing in light blue. (**D**) Histo-morphological analysis of the spleen after one month of exposure (open bars) or eight months or exposure (hatched bars). The ratio of the red pulp to the spleen surface showed no change as a function of the group of exposure or to the duration of exposure (two-way ANOVA test). (**E**,**F**) Immuno-histological analysis of HMOX and CD36 expression in the spleen of exposed animals after one month of exposure. (**E**) Representative HMOX staining showing nuclear staining mainly in the germinal center (arrows, upper panel) and representative CD36 staining showing a homogeneous cytoplasm staining in the red pulp (arrows, lower panel) in a control animal after one month of exposure. (**E**) Immuno-histo-morphological analysis of CD36 staining in the spleen of mice exposed for one month. No significant changes were observed in CD36 expression according to the group of exposure (one-way ANOVA test).

As an alternative hypothesis, it is possible that the decrease seen in RBC numbers was due to an excessive retention of RBC within the spleen. In order to verify this, the level of mRNA expression of a number molecules was measured, including CD36, an adhesion molecule implicated in RBC adhesion to macrophages, Heme oxygenase-1 (HMOX-1), which is implicated in heme recycling, divalent metal transporter 1 (DMT-1) and the iron regulated transporter (IREG), two molecules implicated in iron transport. As shown in Fig. 5A,B, changes were observed in the relative expression of CD36 mRNA and DMT1 mRNA in both HTO and TAA-exposed groups after one month of exposure. Similar results were observed for HMOX-1 and IREG (data not shown). These changes are consistent with the hypothesis that the slight anemia observed in the RBC counts could be regulated through the reduced capture of red blood cells in the spleen after one-month exposure. Interestingly, after an 8-months exposure, the relative expression of CD36, HMOX, DMT1 and IREG normalized - consistent with the normalization seen in the number of RBC in the blood. The results confirm that the RBC retention in the spleen after one month facilitated the regulation of RBC numbers. In order to confirm this, histological and immuno-histological analysis of CD36 and HMOX expression were performed in the spleen. Figure 5C shows the typical morphology of spleen after one month of exposure and Fig. 5D shows the absence of modification in the ratio of red pulp, the site of RBC retention, to total surface in the spleen. This shows that there is no gross histological modification of the spleen after exposure to either HTO or TAA because of the noted decreases in RBC. CD36 and HMOX1 staining showed a normal pattern of expression in the spleen (Fig. 5E,F) and no changes were seen as a function of group or duration of exposure. The discrepancy between these histological results and changes seen in mRNA expression may be a function of an insufficient sensitivity of the histological analyses. Overall, the results of the spleen analysis suggested that the decrease in RBC number observed after one month of exposure was regulated through a decreased retention of RBC within the spleen and that this retention returned to normal after 8 months of exposure - at the time when the RBC numbers returned to normal compared to the control group. This confirmed that the RBC decrease observed at one month was not linked to a change in RBC retention within the spleen but rather that the changes observed in the spleen are linked to a compensation mechanism for the RBC decrease. Other hypotheses that could explain the observed mild anemia were then explored.

### Plasma iron and protein measurements

**Figure 6:**
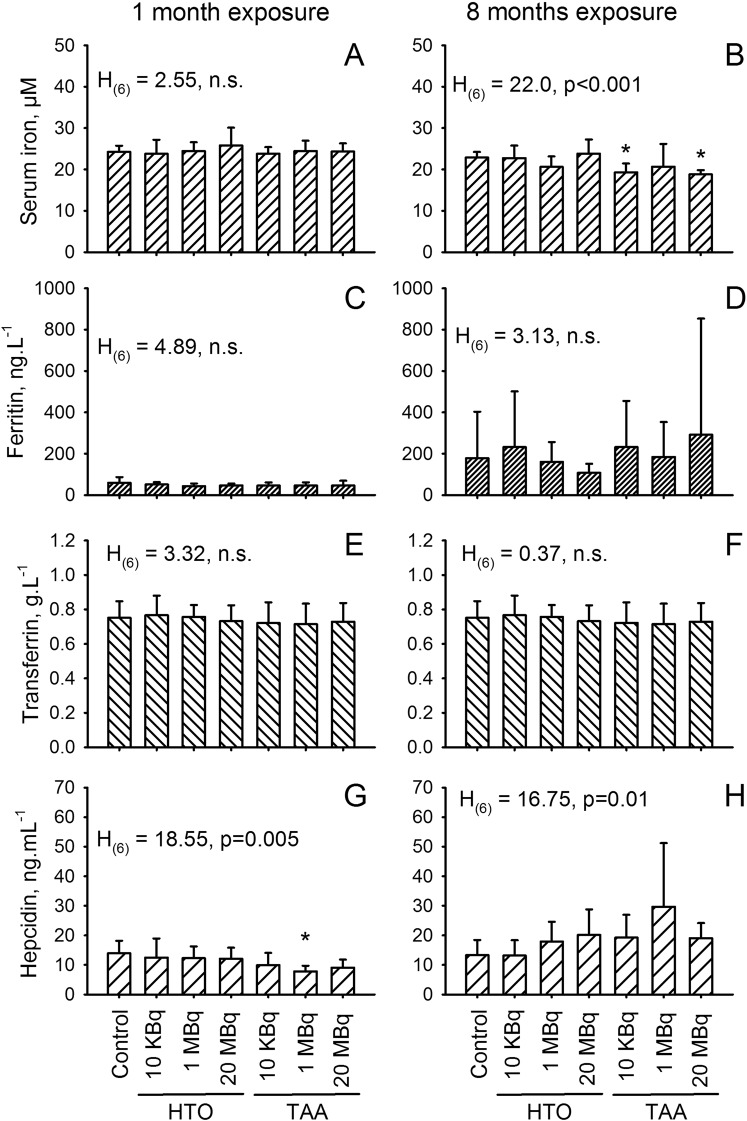
Plasma measurements of (**A**,**B**) serum iron; (**C**,**D**) ferritin; (**E**,**F**) transferrin and (**G**,**H**) hepcidin in animals exposed for one month (**A**,**C**,**E**,**G**) and for eight months (**B**,**D**,**F**,**H**). Results are presented as a mean ± SD of ten animals and results of the one-way anova analysis is indicated for each parameter and for each duration of exposure. A significant difference *versus* the control group was observed for *p < 0.05, using a multiple comparison procedure (Dum’s method). Since the control group served as the reference for the relative gene expression at each duration of exposure, it was not possible to compare data from the one-month exposure with data from eight-month exposure.

Another pathway that may be implicated in the development of an anemia is a deficit in iron metabolism. Thus, iron and proteins associated with iron metabolism were measured, namely ferritin, transferrin and ceruloplasmin in the plasma of exposed and non-exposed animals. Results (Fig. 6B) showed changes in the concentration of iron plasma concentration after 8 months of exposure to TAA in the 10 kBq.l^−1^ and the 20 MBq.l^−1^ group, and (although non-significant) in the 1 MBq.l^−1^ group. By contrast, no change was observed after one month of exposure, either in the HTO or in the TAA groups of exposure (Fig. 6A). This is consistent with the regulation of the one-month RBC decrease by the increased half-life of RBC and the reduced MCCH at 8 months. However, proteins implicated in iron transport in the blood, i.e. ferritin (Fig. 6C,D), transferrin (Fig. 6E,F) and ceruloplasmin (data not shown) were not modified by exposure, regardless of the duration of exposure. These results strongly suggest that the RBC decrease observed after one month which was compensated at eight months through an increased RBC life span and size results in a defect in iron, in association with TAA exposure, but not with HTO exposure.

Interestingly, when looking at hepcidin, a molecule strongly implicated in the regulation of iron metabolism^36,37^, we observed a decreased concentration of hepcidin in the plasma after one month of exposure (Fig. 6G) and an increase after 8 months of exposure (Fig. 6H) both mainly in the TAA-exposed groups. Hepcidin is negatively regulated by erythroferrone (ERFE) in the liver^38,39^, and Hepcidin level in the blood regulates the bioavailability of iron in the blood, especially by increasing uptake of iron by the intestine^40^. We attempted to measured ERFE in the plasma, but all measurements were below the detection limit (0.15 ng/ml) indicating that the ERFE concentration in the blood was normal^39^. This suggests that the defect in iron, mainly observed after eight months of exposure, originates either in the iron-regulatory role of the liver or in the ability of intestine to absorb iron from the food.

### Iron metabolism in the liver and the intestine

**Figure 7:**
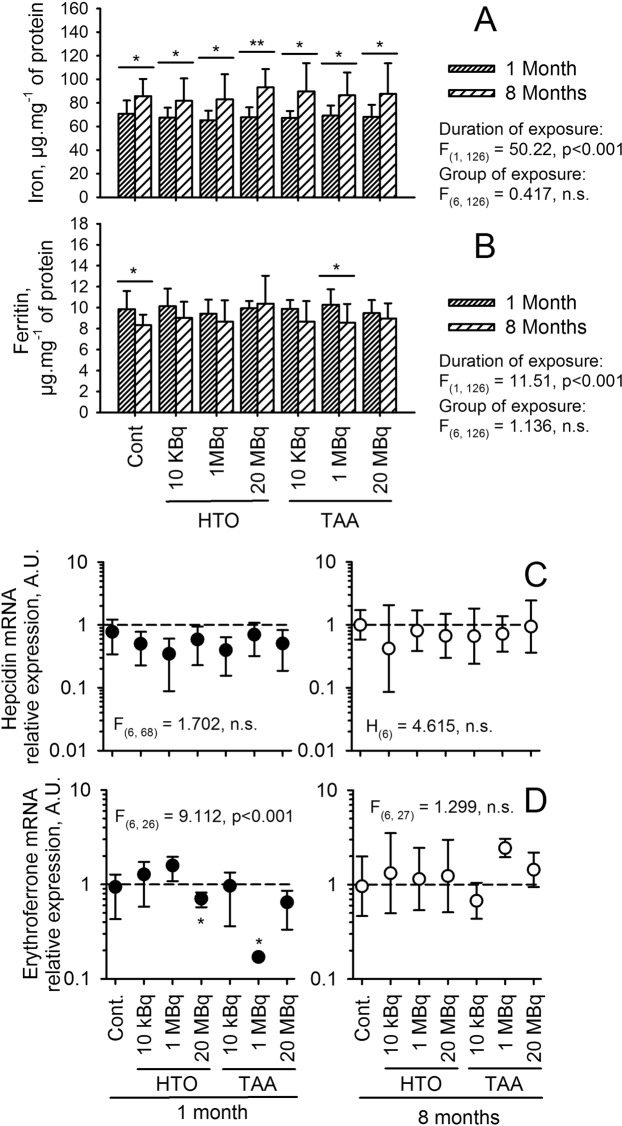
Iron metabolism in the liver. (**A**) Iron concentration and (**B**) ferritin concentration in liver extracts, expressed as mean ± SD of ten animals per group. For iron concentration (**A**), a two-way anova analysis showed a significant difference with duration of exposure (which relates to an age effect) (F_(1, 126)_ = 50.2, p > 0.001) but not with group of exposure (F_(6, 126)_ = 0.42, n.s.). For Ferritin concentration (**B**), difference according to the duration of exposure is significant (age-related effect) (F_(1, 126)_ = 11.5, p > 0.001), but not with the group of exposure (F_(6, 126)_ = 1.14, n.s.). Differences between 1 and 8 months of exposure within the same group of exposure are indicated for *p < 0.05 and **p < 0.001. (**C**,**D**) mRNA expression of hepcidin (**C**) and erythroferrone (**D**) after one (left panel) or eight months (right panel) of exposure. Results are presented as a mean ± SD of ten animals and results from the one-way anova or the anova on ranks analysis are indicated for each graph. Significant differences with the control, using a multiple comparison procedure are indicated for *p < 0.05. Since the control group served as the reference for the relative gene expression at each duration of exposure, it was not possible to compare data from the one-month exposure with data from eight-month exposure.

We then explored the iron metabolism in the liver. As a first step, we looked at the iron concentration in liver extracts. Results showed that iron concentration is not modified by the group of exposure, either after one month or after eight months of exposure (Two-way anova, F_(6, 126)_ = 0.417, n.s.) (Fig. 7A). Also, no change in ferritin concentration was observed as a function of the group of exposure (Two-way anova, F_(6, 126)_ = 1.136, n.s.) (Fig. 7B). In comparison, a significant increase in iron concentration was observed after 8 months of exposure compared to after one-months exposure (F_(1, 126)_ = 50.22, p < 0.001) in all exposure groups, including the control, and a significant decrease in ferritin concentration was also observed by exposure duration (F_(6, 126)_ = 11.51, p < 0.001). However, these changes appeared to be age-related, since these changes in iron and ferritin concentrations were also observed in non-exposed control groups. In order to confirm the absence of changes in iron metabolism, the expression of mRNA coding hepcidin, ERFE (Fig. 7C,D), DMT1, IREG, ferritin chains H and L and transferrin receptor 1 and 2 was also checked. However, no significant changes in the expression of these mRNAs were observed, apart from ERFE which showed a decreased expression after one month of exposure to TAA but not after 8 months of exposure or after exposure to HTO (Fig. 7D). This decreased expression of ERFE in the liver may explain the decreased concentration of hepcidin in the plasma observed after one month of exposure. Also, the return of ERFE expression to the control values after eight months of exposure might also explain the increased concentration of hepcidin in the plasma. It is suggested that the negative regulation of ERFE expression is a consequence of the RBC decrease observed after a one-month exposure, suggesting that the RBC decrease induced by exposure to TAA does not stem from a defect of iron regulation by the liver.

**Figure 8:**
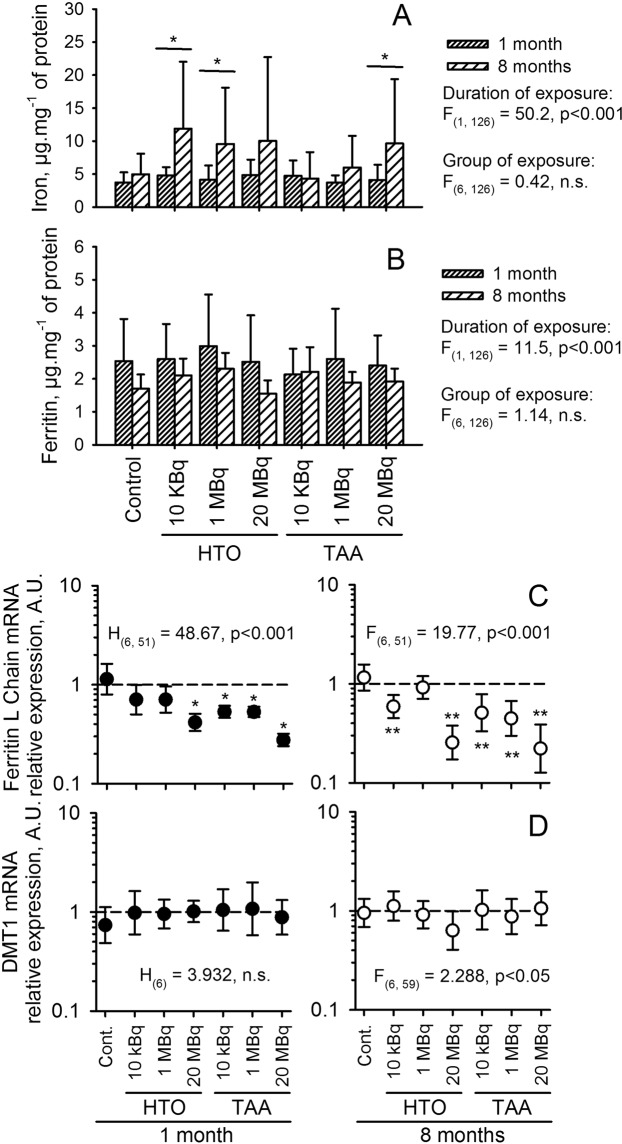
Iron metabolism in the intestine. (**A**) Iron concentration and (**B**) ferritin concentration in intestinal mucosa extracts, expressed as a mean ± SD of ten animals per group. For iron concentration (**A**), a two-way anova analysis showed a significant difference with the duration of exposure (which relates to an age effect) (F_(1, 126)_ = 50.2, p > 0.001) but not with the group of exposure (F_(6, 126)_ = 0.42, n.s.). For the ferritin concentration (**B**), difference according to the duration of exposure is significant (Age-related effect) (F_(1, 126)_ = 11.5, p > 0.001), but not with the group of exposure (F_(6, 126)_ = 1.14, n.s.). Differences between 1 and 8 months of exposure within each group of exposure, tested with a multiple comparison method, are indicated for *p < 0.05 and **p < 0.001. (**C**,**D**) mRNA expression of ferritin L (**C**) and DMT1 (**D**) after either one (left panel) or eight months (right panel) of exposure. Results are presented a mean ± SD of ten animals and results from the one-way anova or the anova on ranks analysis are indicated for each graph. Significant differences with the control, using a multiple comparison procedure are indicated for *p < 0.05 and **p < 0.001. Since the control group served as the reference for the relative gene expression at each duration of exposure, it was not possible to compare data from one-month exposure with data from eight-month exposure.

The metabolism of iron in the intestine was explored, to test for iron capture in the alimentary tract. Measurement of iron concentration in mucosal extracts showed no changes as a function of exposure group (two-way anova, F_(6, 126)_ = 0.42, n.s.), but showed a significant increase with exposure duration (F_(1, 126)_ = 50.2, p < 0.001) (Fig. 8A). Significant differences were found between one month and 8 months in the HTO 10 kBq.l^−1^ and 1 MBq.l^−1^ and TAA 20 MBq.l^−1^ groups of exposure. This result suggests an increased accumulation of iron in the intestinal mucosa. Similarly, no change in ferritin concentration according to the group of exposure was evident (F_(1, 126)_ = 1.14, n.s.) while significant decreases in ferritin concentration were observed with duration of exposure (F_(1, 126)_ = 11.5, p < 0.001) (Fig. 8B).

In order to confirm these changes, the expression of mRNA coding DMT1, IREG, HMOX, HIF-2α, ferritin chains H and L and transferrin receptor 1 was checked. No change in expression was observed in all of these genes excepted for ferritin L chain (Fig. 8C,D) for which a strong decrease in the mRNA expression was observed both after one month (F_(6, 51)_ = 48.67, p < 0.001) and after 8 months of exposure (F_(1, 51)_ = 19.77, p < 0.001) (Fig. 8C). Moreover, the decreased expression of mRNA encoding ferritin was observed mainly in the TAA exposed groups, although some changes were also observed in the HTO exposed groups. This is consistent with the decreased concentration of ferritin protein in intestinal mucosal extracts and suggests that the RBC decrease observed in animals exposed to TAA could be due to a defect in iron capture in the intestine.

**Figure 9:**
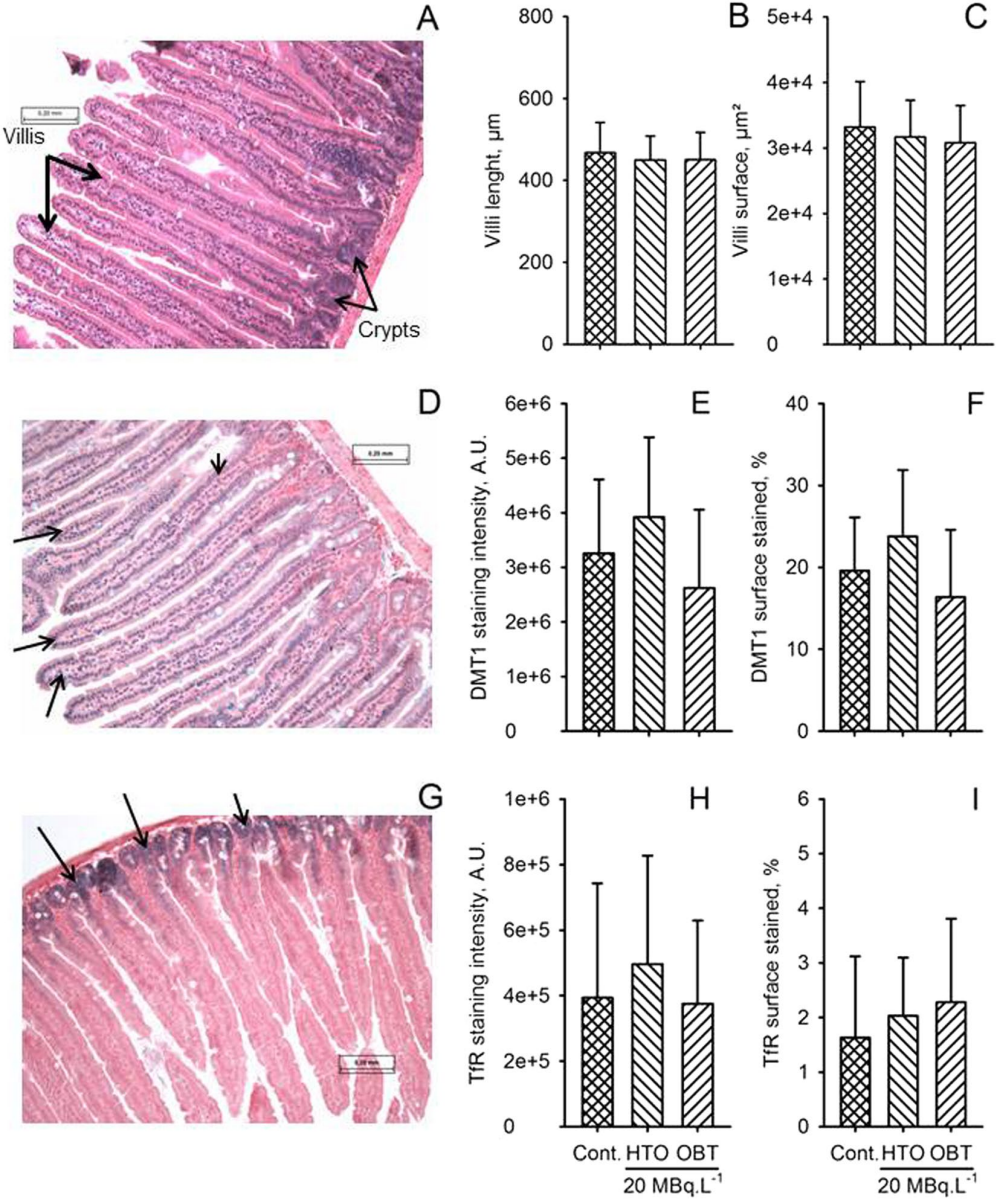
Histological and immuno-histological analysis of the intestine. (**A**) HES staining of intestine section, showing a normal appearance of villi (large arrow) and crypts (thin arrows). Cytoplasm is stained in pink and nuclei are stained in dark blue. (**B**,**C**) Morphological analysis of villi, using villi height (**B**) and villi surface for control HTO 20 MBq.l^−1^ and TAA 20 MBq.l^−1^ groups. No significant differences were observed between controls and exposure groups (Student’s t test). (**D**) Immuno-histological staining of intestine for DMT1 expression, appearing in dark blue (arrows), with nuclear fast red counterstaining. DMT1 is mainly expressed in the nucleus of cells in the villi and is less expressed in the basal part of the villi and in the crypt. (**E**,**F**) Analysis of DMT1 staining, showing that the staining intensity (**E**) and the surface stained (**F**) are not significantly modified by the exposure to either HTO or TAA as compared to the control group (Student’s t test). (**G**) Immuno-histological staining of the intestine for transferrin receptor (TfR), showing that the TfR is mainly expressed in the crypts and in the basal part of the villi. Staining for TfR appears in dark blue (arrows) with nuclear fast red counterstaining. (**H**,**I**) Analysis of TfR staining, showing that the staining intensity (**H**) and the surface stained (**I**) are not significantly modified by the exposure to either HTO or TAA as compared to the control group (Student’s t test).

A histo-morphological analysis of the intestine was thus performed to detect gross histological changes, either after one month or after 8 months of exposure, in control, HTO and TAA 20 MBq.L^−1^ groups. This histo-morphological analysis was made on the proximal part of the jejunum, since the absorption of polypeptides and AAs take place mainly in the duodenum and in the proximal jejunum^41,42^. Results of HES staining indicated that the villi length and surface were not modified after one month (Fig. 9A–C) or after 8 months of exposure (data not shown). The expression of two molecules, DMT1 and transferrin receptor (TfR), were also explored. DMT1 showed mostly nuclear staining (Fig. 9D), while TfR was mainly expressed in the intestinal crypts and in the basal part of the villi (Fig. 9G). Again, significant changes in neither the staining intensity, nor in the percent of stained surface was observed between the control group and the two exposure groups tested after one-month exposure (Fig. 9E,F,H,I). These results suggest that the molecular changes detected for ferritin and iron are not linked to major histological changes in the intestinal wall. This confirms and extends previous results indicating the absence of major changes in apoptosis or in proliferation within intestinal epithelial cells, regardless of duration or group of exposure^26^.

### Effect of external gamma irradiation on RBC and iron metabolism

**Figure 10:**
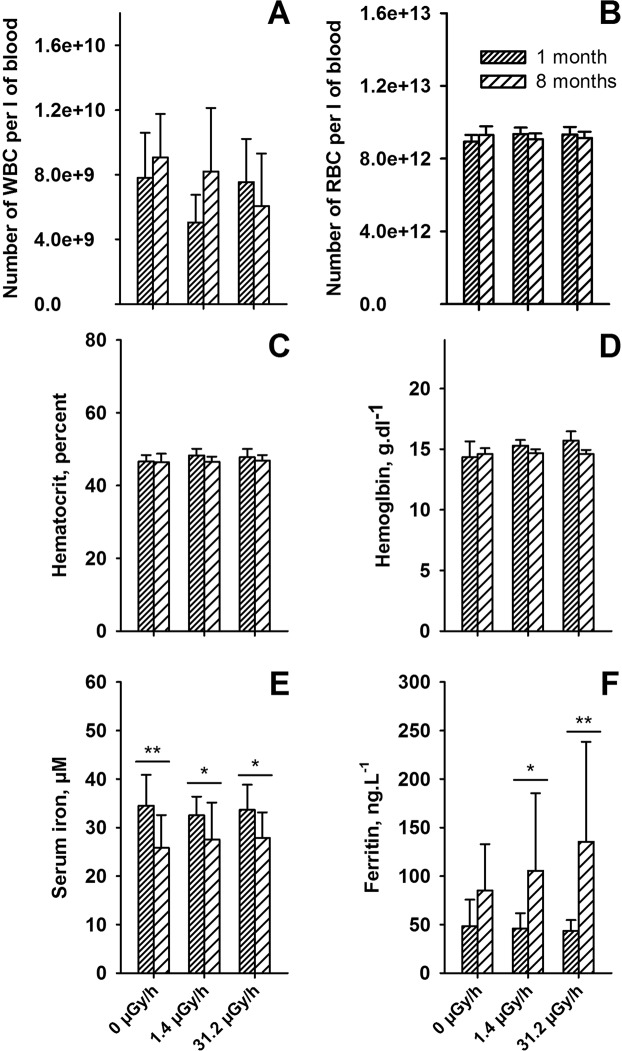
Effect of external gamma irradiation at two dose rates, 1.4 µGy.h^−1^ and 31.2 µGy.h^−1^ equivalent to internal exposure to 1 MBq.l^−1^ and 20 MBq.l^−1^ on blood parameters. (**A**) White blood cell (WBC) numbers; (**B**) red blood cell numbers; (**C**) hematocrit; (**D**) hemoglobin concentration; (**E**) serum iron concentration; (**F**) Ferritin concentration. Significant differences according to the duration of exposure were observed, using a two-way anova test, for serum iron concentration (F_(1, 64)_ = 20.84, p < 0.001) and for ferritin concentration (F_(1, 58)_ = 19.05, p < 0.001), but not as a function of the dose rate applied (F_(2, 64)_ = 0.093, n.s. for iron and F_(1, 58)_ = 0.90, n.s. for ferritin, respectively).

In a parallel experiment, groups of mice were irradiated at dose rates matching those employed for the tritium irradiations. Doses were calculated using data generated by previously described biokinetic studies^43–45^. The dose rates used were 1.4 µGy.h^−1^ (the lowest practical dose rate achievable in the exposure hall) and 31.2 µGy.h^−1^ corresponding respectively to the 1MBq.l^−1^ and 20 MBq.l^−1^ TAA concentrations in drinking water. Results of blood cell counts showed no significant changes in either WBC or RBC numbers, after either 1 month or 8 months of exposure (Fig. 10A,B). Accordingly, no significant changes were observed in hematocrit or in hemoglobin concentration (Fig. 10C,D), demonstrating the absence of anemia in animals exposed to external gamma irradiation. In order to confirm this result, blood parameters in relation to iron metabolism were measured. No changes were observed in ceruloplasmin concentration, or in transferrin concentration in the plasma of irradiated animals compared to controls, irrespective of the duration of exposure. However, a decrease in iron concentration (F_(1, 64)_ = 20.84, p < 0.001) and an increase in ferritin concentration (F_(1.58)_ = 19.05, p < 0.001) were observed with the duration of exposure (Fig. 10E,F), but not with the dose rate. These results indicated that the changes seen were most likely due to animal aging. This is suggested by the observed decrease in ferritin concentration in the control group after 8 months compared to that seen in the one-month control group. Overall, these results indicated that tritium equivalent (either in the form of HTO or TAA) external gamma irradiation did not induce a change in either hematologic parameters or iron metabolism.

## Discussion

Iron metabolism involves a complex interplay between the physiology of several organs including spleen, kidney, liver, bone marrow and intestine^46^. The initial observation of RBC number decrease after one month of exposure with subsequent compensation by increases in RBC volume (and thus life-span) after 8 months of exposure is clearly linked to a decreased availability of iron in the blood, mainly visible after 8 months of exposure. Since the decrease in RBC number was in the range of 4–6% and the decrease in hemoglobin concentration was in the range of 4–5.2%, this represented a limited anemia.

**Figure 11:**
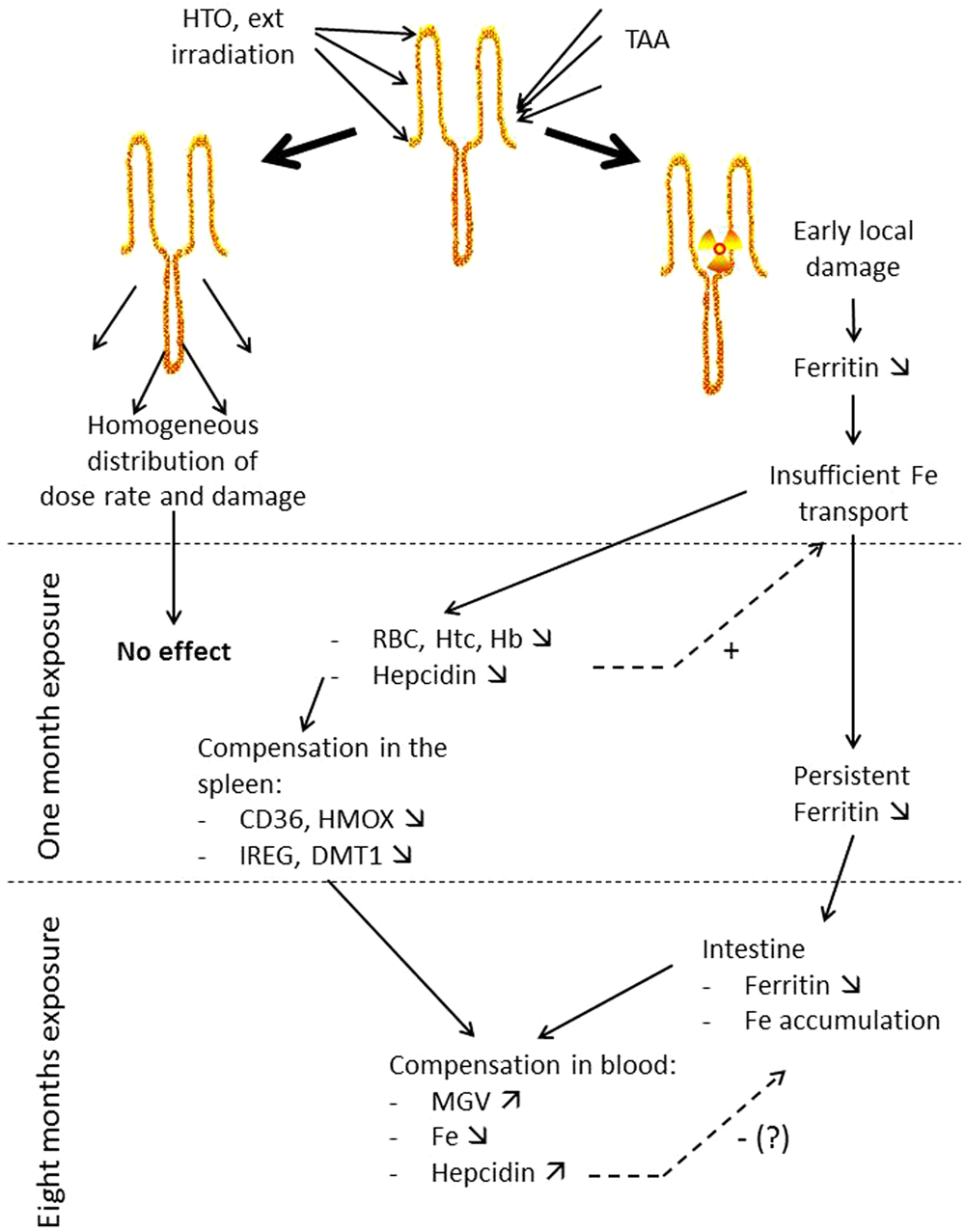
Schematic view of the results obtained after one or eight months of exposure to TAA, HTO and external irradiation and of the hypothesis possibly explaining the observed results. The hypothesis proposed is that local damages produced by TAA in the intestine induced a decreased ferritin expression which resulted in a decreased ability of iron transport through the intestinal barrier. As a result of insufficient iron availability, a decrease in RBC is observed and changes in iron metabolism in the spleen after one month of exposure. Due to both the persistent damage in the intestine with iron accumulation, this leads to iron deficit in the blood at 8 months. The increase in MVG thus appears as a compensation mechanism for the reduced number of RBC at one month. Hepcidin low concentration in the blood appears as a positive regulator of iron capture by the intestine at one month, not effective due to the persistent damage with the continuous presence of TAA. Such local damage is not induced by HTO or external irradiation due to the homogeneous distribution of radiation dose and damages.

Changes observed after one month of exposure in the spleen are directly linked to an adaptive mechanism to the decreased number of RBC, through a reduced retention of RBC within the spleen, therefore increasing the life span of RBC. By contrast, the decreased hepcidin concentration in the blood is most likely linked to a regulation loop of RBC numbers through interaction between ERFE and Hepcidin^47^. A decreased hepcidin concentration is correlated with an increased uptake of iron in the intestine^48,49^ and is possibly due to an increased ERFE release either in the bone marrow or in the liver^39^. Unfortunately, we were unable to test for ERFE expression or concentration in the bone marrow, and ERFE in the plasma was below 0.15 ng.ml^−1^, indicating a normal ERFE concentration in the blood^39^. The fact that hepcidin is decreased in the blood both after one month of exposure and that level of hemoglobin is compensated through increased life span of RBC suggests that the RBC decrease could originate either in a defect in iron capture in the intestine, or in an increase in iron elimination by the kidney. Previous results indicated that kidney function appears normal as assessed with urine biochemical parameters; however a definite increase in inflammation and oxidative stress were observed^26^. The normal renal function was consistent with our results showing a normal expression of EPO mRNA and a normal EPO level in the blood. By contrast, a strong decrease of ferritin mRNA expression was observed in the intestine, both after one month and 8 months of exposure, in association with iron accumulation in the intestinal mucosa. This suggests that the exposure to TAA induces a persistent defect in iron capture and/or transport in the intestine, which results in a reduced availability of iron and decreases in the numbers of RBC (Fig. 11). In this context, the increased concentration of hepcidin in the plasma after 8 months of exposure is intriguing. In fact, such an increase, although limited, may act as an amplification loop of the decreased ability of the intestine to upload iron. This slight increase in hepcidin at eight months of exposure is possibly linked to the return to control levels of ERFE expression in the liver.

Since the TAAs selected for this study are non-essential AA, they are diluted in the amount of AA present in the diet. Taking into account that the chow contains 18% of crude proteins, that the vertebrate proteins contain as a mean 9% of alanine, 7.5% of glycine and 4.6% of proline^29^ and that an adult mouse eat approximately 3.2 g of chow per day^27,28^, it is possible to estimate the dilution factor of TAA added in drinking water for the highest activity (20 MBq.l^−1^) by the AA content in chow. Results indicate a dilution factor in the range of 1.10^−7^ to 1.10^−8^. It is thus very unlikely that the addition of these quantities of TAA to the diet of animals may induce a chemical toxicity or may increase the effect of proline on metabolic activities^30^. In turn, this strongly suggests that the observed effect of TAA on iron metabolism is due to the radiological effect of tritium incorporated in these AAs.

Interestingly, the effect of tritium on iron metabolism was mainly observed with exposure to TAA, with almost no changes observed with tritium exposure in the form of HTO and no change observed with external irradiation at an equivalent dose rate. Moreover, the effect of TAA exposure was dose dependent on RBC parameters, CD36 and DMT1 mRNA expression in the spleen after one-month’s exposure and on ferritin mRNA expression in the intestinal mucosa both after one or eight months exposure. These results underline the importance of the tritium speciation. Actually, HTO is distributed homogeneously in the body, mostly in the exchangeable water compartment, as previously described^44^. As a result, the dose distribution is also quite homogeneous, especially if one considers the very short range of beta particles emitted by tritium disintegration (<6 µm)^6^. By contrast, amino acids may be distributed more heterogeneously, since they enter cell cytoplasm and participate in cell metabolic processes. For example, the intestinal epithelium, and especially the basal part of the villi where amino acids are absorbed by enterocytes^50^, may concentrate TAA before transport into the blood. Moreover, the AAs are absorbed mainly by the duodenum and the upper part of the jejunum^42^. Lastly, a significant proportion of AAs (up to 50% depending on the AA) are catabolized by enterocytes^41^. Therefore, at the level of micro-dosimetry, one cannot exclude that local concentrations of TAA may occur in the enterocytes, thus contributing to the appearance of cell damage. Such a local concentration of TAA might not be observed in biokinetics experiments when measuring tritium content of the whole intestine, as previously described^44^. We hypothesize that in the intestine, this cell damage manifests as a decreased ferritin expression, resulting in defective iron transport in the intestinal epithelium (Fig. 11) and a mild anemia after a one-month’s exposure. Such a blockade of iron capture by mucosal cells has already been described^51^. Thus, all other observed changes appeared because of this defect in iron transport in the intestine. However, one cannot exclude the possibility that damage was produced by the local concentration of TAA elsewhere in the organism. Nevertheless, we think that this is unlikely, since analyses of toxicity in various tissues of the same mice used in this study did not reveal any tissue or metabolic major specific effects^26^. It would be of interest to test this hypothesis using other forms of OBT with a different metabolic behaviour, such as polysaccharides or lipids. One can hypothesize that different biological effects should be observed depending upon the local accumulation of each of these different OBT forms. Such observations would be consistent with our hypothesis to explain the indirect effect of TAA ingestion on iron metabolism. Interestingly, in previous studies on germ cells in the mouse, it was shown that some forms of OBT such as L-lysine and tritiated nucleosides are more efficient in inducing dominant lethal mutations than tritium as HTO^52^, supporting our hypothesis specifying a specific target for TAA in the intestine.

In addition to the above it would be of interest to examine the impact of extending the duration of exposure. This is so, because the eight months exposure employed by the study, plus the two-month age at start, corresponds to 10 months, which is much less than the life expectancy of the mouse model. In contrast, human exposures may last for years or decades, e.g., for populations living in the vicinity of nuclear power plants. Moreover, we observed larger individual variations in several parameters after eight months of exposure than after one-month’s exposure, including for EPO and Flt3-L concentrations, hepcidin and ferritin in the plasma, iron in the liver and in the intestine. We also observed an effect of duration of exposure on several parameters such as Iron and ferritin concentration in the liver and in the intestine. Therefore, a longer duration of exposure might show larger or even different effects of tritium exposure on either iron metabolism or on other physiological systems.

Our results support the current regulations related to the level of tritium in drinking water^2,4^. The lowest concentration of tritium used in this study, 10 kBq.l^−1^, corresponds to the WHO’s recommendation level. At this concentration, no biological effects due to HTO were observed. Even at concentrations 100-fold higher almost no effects of HTO ingestion were observed. Thus, the recommended level of 10 kBq.l^−1^ appears to be sufficiently protective for HTO. For of TAA, the situation is somewhat different. Indeed, limited, but significant biological effects were observed at 10 kBq.l^−1^ and biological effects of larger amplitude were observed at 1 MBq.l^−1^ with a clear dose response. However, the probability of exposure is low because of the low percentage of OBT forms in food and its almost complete absence from drinking water. In most cases, OBT forms of tritium represent <30% of all tritium forms in plants and animals^8^ but <1% in drinking water. Moreover, the three TAA used in this study may represent less than 0.1% of all OBT forms and probably much less. Thus, TAA in drinking water may represent far less than 0.001% of all chemical forms of tritium in drinking water. This means that a TAA concentration of 10 kBq.l^−1^ in water should correspond to an overall concentration of tritium of <1000 GBq.l^−1^. Since in the present study effects of a 20 MBq.l^−1^ HTO concentration produced only limited effects and recognizing that similar results were obtained in other studies in the same model^26,43,45,53^, it appears that the occurrence of biological effects attributable to an OBT form of tritium at the regulatory concentration level in drinking water is highly unlikely. Therefore, the current regulations about tritium in drinking water appear to be defendable as sufficiently protective.

## Material and Methods

### Animals, exposure and organ treatment

Male C57BL/6J 7-weeks old mice were obtained from Jackson Laboratories (Bar Harbor MN, USA). They were housed, 5 per cage with a 12hrs/day light cycle and received *ad libitum* commercial rodent chow (Charles River Laboratories, Montreal, Canada, rodent chow #5075, 18% crude protein content) and reverse-osmosis water. They were acclimatized for 7 days before starting exposure. All animal procedures were submitted and approved by the Chalk River animal care and welfare committee (Permit # DRF-09-05) and were conducted in accordance with current Canadian regulations.

Exposure of mice to tritium started at 8-weeks of age for either one month or eight months, through a drinking water bottle. Tritium was in the form of either tritiated water (HTO) (CNL, Canada) or in the form of a mixture of three non-essential AA, alanine, proline and glycine with equal activity for each AA (Perkin Elmer, Woodbridge, ON, Canada; specific activity 2.4–3.15 TBq/mmol; 1.3–2.0 TBq/mmol and 1.1–2.2 TBq/mmol respectively), hereafter referred as tritiated amino acids (TAA). For each form of tritium, three concentrations were used, 10 kBq.l^−1^, 1 MBq.l^−1^ and 20 MBq.l^−1^, together with a non-exposed, control group. The final concentration of ^3^H in HTO and OBT water preparation was confirmed by liquid scintillation counting (Tricarb liquid scintillation analyzer 1900CA, Perkin-Elmer. Concord Ontario, Canada). Assuming a daily water consumption of 5 ml and a mean weight of 20 g during the experiment, this represents a mean ingestion of 2.5 kBq.kg^−1^.day^−1^, 250 kBq.kg^−1^.day^−1^ and 5 MBq.kg^−1^.day^−1^. Mice were randomly assigned to one of these exposure groups.

In a second experiment, groups of mice were non-exposed (control group) or exposed to a low dose rate external gamma irradiation with a cobalt-60 source at the CNL Chalk River animal facility. The dose rates were set for the irradiation dose to be equivalent to the internal tritium dose at the two highest concentrations, i.e. 1 MBq.L^−1^ and 20 MBq.L^−1^, and according to the tritium concentration measured in animals^43–45^. The formula for calculation of absorbed dose rate is the following:

$${\rm{Xt}}={\rm{D}}\times {\rm{P}}\times {\rm{b}}\times {\rm{E}}\times {\rm{T}}$$

were Xt is the absorbed dose to the mouse per unit of time (t), D is the concentration of tritium in drinking water (in Bq.L^−1^), P is the percent of tritium in tissues relative to that in drinking water, b is the mean energy of beta particles from tritium (5.7 × 10^−3^ MeV), E is the conversion factor from MeV to Joules (1.6021 × 10^−13^J/Mev, T is the time t in seconds. For the one-month period, averaged P value was derived from individual time intervals within the 30-day period. For the 8 months exposure, a dose from the first month was summed with the dose from the subsequent 7 months. Resulting dose rates corresponding to 1 MBq.L^−1^ and 20MBq.L^−1^ of tritium in drinking water were 1.4 µGy.h^−1^ and 31.2 µGy.h^−1^ respectively and the exposure lasted for either one or eight months. The exposure to external irradiation was continuous except for one short period every day used for animal care and a longer period every other week for cage change. In the total experimental period, the mean stoppages in irradiation exposure were 37 minutes per day and 2.6 hours every other week. Cumulated dose was controlled with thermo-luminescent dosimeters.

After one or eight months of exposure, animals were anesthetized by isoflurane inhalation. Blood was obtained by intra-cardiac puncture using a heparinized syringe (Sanofi-Aventis, Gentilly, France) and animals were euthanized by cervical dislocation. Spleen, kidneys, small intestine, liver and femurs were harvested. Blood was used immediately for numeration and differential using a MS5-vet automated device (Melet-Schlossing, Osny, France), before being centrifuged for 10 min. at 400 g. Plasma was harvested and frozen for later use. A 1-cm length sample of the upper part of the jejunum was excised and fixed in buffered formalin for 24 hrs. The remaining jejunum was scraped in order to isolate intestinal mucosa, which was then subsequently frozen. Remaining organs were separated in two parts, one being frozen, the other one fixed in formalin for 24 hrs.

### Gene expression analysis

Total RNA was isolated from spleen, liver, kidney and intestinal mucosa using a ribolyser (Bertin technologies, Montigny le Bretonneux, France) and trizol reagent (Sigma-Aldrich, St Quentin Fallavier, France). RNA was then purified using an affinity column-based kit (RNeasy totalRNA isolation kit; Quiagen, Courtaboeuf, France) according to the manufacturer’s recommendations. RNA concentration and integrity were checked by OD measurement at 230 nm and the 260 nm/280 nm OD ratio (Thermo Scientific nanodrop 1000, Labtech, Palaiseau, France). One microgram of total mRNA was reverse transcribed with random hexamers and a high-capacity cDNA reverse transcription kit was used according to the manufacturer’s recommendations (Applied biosystems, Courtaboeuf, France). Gene expression was measured by real-time polymerase chain reaction (PCR). cDNA (5 or 10 ng) was amplified in duplicate using SYBR Green PCR master mix (Applied biosystems). Forward and reverse primers used in this study (obtained from Life Technologies, Cergy-pontoise, France) and corresponding amplification efficiencies are indicated in Table 1. PCR products were amplified and detected with the Quant studio 12 (Applied Biosystem). The resulting fractional cycle number of the threshold (Ct) was used for transcript quantification. Expression levels of each sample were normalized to the geometric mean expression of three reference genes namely glyceraldehyde 3-phosphate deshydrogenase (GAPDH), 60 S ribosomal protein L41 (RPL41) and Hypoxanthine-guanine phosphoribosyltransferase (HPRT) genes. Expression relative to the control group was calculated for each gene by the 2^−ΔΔCt^ method as previously described^54^.

**Table 1:** List of primers used in this study. All primers were obtained from Life sciences (Cergy-Pontoise, France).

Name	Forward primer	Reverse primer	Efficiency % (1)
CD36	GAG CAA CTG GTG GAT GGT TT	GCA GAA TCA AGG GAG AGC AC	109.0
Heme oxygenase-1 (HMOX-1)	CCA GAG TGT TCA TTC GAG CA	CAC GCA TAT ACC CGC TAC CT	101.9
Divalent metal transporter 1 DMT1)	TCC TCA TCA CCA TCG CAG ACA CTT	TCC AAA CGT GAG GGC CAT GAT AGT	104.8
Iron regulated transporter (IREG)	TGT TGT TGT GGC AGG AGA AA	AGC TGG TCA ATC CTT CTA AT	100.0
Erythroferrone (ERFE)	TCT ACA GGC AGG ACA CTA CAC	CTG TCA CCA CTC TGC TTG GTA	103.3
Ferritin L	CGT CTC CTC GAG TTT CAG AAC	CTC CTG GGT TTT ACC CCA TTC	102.4
Transferrin 1	AGA GGC GCT TCC TAG TAC TCC	CTT GCC GAG CAA GGC TAA AC	101.3
Transferrin 2	CCA AGA AAC CCA GAG ACC TGT	GAC CTG CAG CTG TCA AAG CC	117.1
Hepcidin	CAT TGC GAT ACC AAT GCA GAA GA	GGA TGT GGC TCT AGG CTA TGT T	104.1
GAPDH	AGC TTG TCA TCA ACG GGA AG	TTT GAT GTT AGT GGG GT CTC G	102.9
Rpl-41	GCC ATG AGA GCG AAG TGG	CTC CTG CAG GCG TCG TAG	98.9
HPRT	GAG GAG TCC TGT TGA TGT TGC CAG	GGC TGG CCT ATA GGC TCA TAG TGC	99.2

(1): The efficiency was calculated using cDNA obtained from the spleen of control animals, excepted for EPO, which was tested using cDNA obtained from the kidney.

### Plasma protein measurement through multiplex

A ten-plex assay was set up in order to simultaneously measure 10 different cytokines in the plasma. For granulocyte-colony stimulating factor (G-CSF), granulocyte-macrophage-CSF (GM-CSF), interleukin-3 (IL-3), IL-6 and mastocyte-CSF (M-CSF) detection, commercially available beads were used (Bio-Rad, Marnes la coquette, France). For erythropoietin (EPO), stem cell factor (SCF), stromal cell derived factor-1 (SDF-1), FMS like tyrosine kinase 3 ligand (Flt3-l) and thrombopoietin (TPO) specific beads were developed using specific antibodies (all from R&D system, Abingdon, UK), uncoupled beads and a coupling kit (All from Bio-rad) according to the manufacturer’s recommendations. The range in which a linear response is obtained, specificity and absence of cross-reactivity of these beads were then assessed using recombinant mouse cytokines (all from R&D systems) before mixing the ten beads in a single assay. Detection limits, defined as 2σ above the mean blank control value were 0.005 ng/ml for EPO, GM-CSF, IL-3, M-CSF and Flt3-l, 0.01 ng/ml for G-CSF and IL-6, and 0.025 ng/ml for SCF, TPO and SDF-1.

### Plasma and tissue parameters measurements

Intestine and liver samples, 30–40 mg each, were subjected to protein extraction using a mammalian cell lysis kit (Sigma-Aldrich) and following the manufacturer’s instructions. Total protein concentration was then measured using the Bradford reagent (Bio-Rad). Iron, ceruloplasmin, transferrin and ferritin concentrations were measured either in these protein extracts or in plasma with a biochemistry apparatus (Konelab 20, Thermo Fischer scientific, Villebon sur Yvette, France) using commercially available kits and following manufacturer’s instructions (Thermo Fischer scientific).

### Protein measurement in tissues

ELISA kits for the detection of hepcidin and erythroferrone in protein extracts from tissues were used according to the manufacturers’ recommendations (Cloud-clone, Houston, TX and LSBio Inc., Seattle, WA, respectively).

### Histological analysis

Once fixed in formalin, tissue samples were embedded in paraffin. 5 µm tissue sections were prepared and fixed on polysin-treated slides (VWR, Fontenay sous bois, France). After rehydration in successive baths of xylene and decreasing concentrations of ethanol, tissue sections were stained with hematoxilin-eosin-safran (HES) and mounted in a Prisma automated staining apparatus (Sakura Finetek, Villeneuve d’Ascq, France). Stained sections were then analyzed with a microscope using Histolab software (Microvision instruments, Evry, France). For each animal, two different spleen sections were used to measure the surface of analysis and of germinal centers. The red pulp surface was then obtained by calculating the difference between the two measured surfaces.

### Immuno-histological analysis

Once rehydrated, tissue sections were heated in the presence of Tris-EDTA buffer pH = 9 (Diagomics, Blagnac, France) to unmask antigens. Membrane permeation was made using a 0.1% Triton X100 solution (Sigma-Aldrich), and endogenous peroxidases were saturated by treatment with 3% hydrogen peroxide solution. Sections were saturated by incubation in the presence of a serum-free protein block (Diagomics), and then incubated in the presence of rabbit anti-mouse primary antibodies at a predefined concentration. Primary antibodies used in this study were rabbit anti-mouse CD36, HMOX-1, DMT1, IREG, transferrin receptor and Ferritin (all from Abcam Ltd, Paris, France). After washing tissue sections in PBS (Life technologies, Cergy-Pontoise, France), they were incubated in the presence of a secondary antibody against rabbit immunoglobulins coupled to horseradish peroxydase (HRP)(Polink-1, GBI labs, Diagomics, Blagnac, France), and staining was revealed by Histogreen solution (Linaris, Eurobio, Les Ulis, France). Counterstaining was performed using nuclear fast red (Vector, Clinisciences, Nanterre, France). After mounting, tissue sections were analyzed with a microscope using Histolab software. For each animal, two tissue sections were analyzed with the following protocol: A surface area was defined in the observed field, and an automated detection of green staining was made, using threshold for size and intensity of the staining. Results were then expressed as staining intensity per µm².

### Statistical analysis

All tests were performed with at least 10 animals per group unless otherwise indicated and results are presented as mean ± standard deviation of the mean (SD). Results were analyzed with either one-way or two-way Anova tests, or with Anova on ranks when normality of the data distribution was not reached. Pair-wise comparisons between groups were performed using the Holm-Sidak method with an adjusted p value for each comparison or using a multiple comparison procedure (Dum’s method). All statistical analyses were performed using Sigmaplot software (Systat software Inc, San José, CA).

## References

[1] UNSCEAR. Annex C: Biological effects of selected internal emitters - Tritium. 241–359 (UNSCEAR, New York, 2016).

[2] WHO. *Guidelines for drinking-water quality, fourth edition*. (World Health Organisation, 2011). 28759192

[3] ICRP. Age-dependent doses to members of the public from intake of radionuclides: Part 1. A report of a Task Group Committee of the International Commission on Radiological Protection. *Annals of the ICRP* **20**, 1–122 (1989). 2633670

[4] Brooks AL, Couch LA, Chad SA (2013). Commentary: what is the health risk of 740 Bq L(−1) of tritium? A perspective. Health physics.

[5] Kocher DC, Hoffman FO (2011). Drinking water standard for tritium-what’s the risk?. Health physics.

[6] ICRU. Linear energy transfer. (ICRU, Washington, 1970).

[7] Eyrolle F (2018). An updated review on tritium in the environment. J Environ Radioact.

[8] Kim SB, Baglan N, Davis PA (2013). Current understanding of organically bound tritium (OBT) in the environment. J Environ Radioact.

[9] Ujeno Y, Yamamoto K, Aoki T, Kurihara N (1986). Tritium Content in Tissue Free Water of Japanese Bodies. Radiation Protection Dosimetry.

[10] Hisamatsu S, Katsumata T, Takizawa Y (1992). Tritium concentration in Akita City diet. Health physics.

[11] Wang B, Watanabe K, Yamada T, Shima A (1996). Effects of beta radiation from organically bound tritium on cultured mouse embryonic mid brain cells. Health physics.

[12] Quan Y, Lin J, Deng B (2019). The response of human mesenchymal stem cells to internal exposure to tritium beta-rays. J Radiat Res.

[13] Harrison J (2009). Doses and risks from tritiated water and environmental organically bound tritium. Journal of Radiological Protection.

[14] Paquet F (2013). The assessment and management of risks associated with exposures to short-range Auger- and beta-emitting radionuclides. State of the art and proposals for lines of research. Journal of Radiological Protection.

[15] Paquet F, Métivier H (2008). Les risques liés aux expositions au tritium sont-ils sous-évalués?. Radioprotection.

[16] Little MP, Lambert BE (2008). Systematic review of experimental studies on the relative biological effectiveness of tritium. Radiation and environmental biophysics.

[17] Schubauer-Berigan MK, Daniels RD, Bertke SJ, Tseng CY, Richardson DB (2015). Cancer Mortality through 2005 among a Pooled Cohort of U.S. Nuclear Workers Exposed to External Ionizing Radiation. Radiat Res.

[18] Zablotska LB, Lane RS, Thompson PA (2014). A reanalysis of cancer mortality in Canadian nuclear workers (1956–1994) based on revised exposure and cohort data. British journal of cancer.

[19] Gillies M, Haylock R (2014). The cancer mortality and incidence experience of workers at British Nuclear Fuels plc, 1946–2005. J Radiol Prot.

[20] Straume T, Carsten AL (1993). Tritium radiobiology and relative biological effectiveness. Health physics.

[21] Yamamoto O (1995). Oral administration of tritiated water (HTO) in mouse. II. Tumour development. Int J Radiat Biol.

[22] Yamamoto O, Yokoro K, Seyama T, Kinomura A, Nomura T (1990). HTO oral administration in mice. I: Threshold dose rate for haematopoietic death. Int J Radiat Biol.

[23] Baglan N, Alanic G, Le Meignen R, Pointurier F (2011). A follow up of the decrease of non exchangeable organically bound tritium levels in the surroundings of a nuclear research center. J Environ Radioact.

[24] CNSC. Tritium releases and dose consequences in Canada in 2006. Report No. INFO-0793, (CNSC, Ottawa, 2009).

[25] Yamamoto O, Seyama T, Itoh H, Fujimoto N (1998). Oral administration of tritiated water (HTO) in mouse. III: Low dose-rate irradiation and threshold dose-rate for radiation risk. Int J Radiat Biol.

[26] Gueguen Y (2018). *In vivo* animal studies help achieve international consensus on standards and guidelines for health risk estimates for chronic exposure to low levels of tritium in drinking water. Environmental and molecular mutagenesis.

[27] Bertho JM (2010). Biodistribution of (137)Cs in a mouse model of chronic contamination by ingestion and effects on the hematopoietic system. Radiation and environmental biophysics.

[28] Synhaeve N, Stefani J, Tourlonias E, Dublineau I, Bertho JM (2011). Biokinetics of (90)Sr after chronic ingestion in a juvenile and adult mouse model. Radiation and environmental biophysics.

[29] Bruice, P. Y. *Organic chemistry*. 4th edition edn, (Pearson education inc., 2004).

[30] Tanner JJ, Fendt SM, Becker DF (2018). The Proline Cycle As a Potential Cancer Therapy Target. Biochemistry.

[31] Mazzaccara C (2008). Age-Related Reference Intervals of the Main Biochemical and Hematological Parameters in C57BL/6J, 129SV/EV and C3H/HeJ Mouse Strains. PloS one.

[32] Nandakumar SK, Ulirsch JC, Sankaran VG (2016). Advances in understanding erythropoiesis: evolving perspectives. Br J Haematol.

[33] Chateauvieux S, Grigorakaki C, Morceau F, Dicato M, Diederich M (2011). Erythropoietin, erythropoiesis and beyond. Biochemical pharmacology.

[34] Bertho, J. M. *et al*. Correlation Between Plasma Flt3-Ligand Concentration And Hematopoiesis During G-Csf-Induced Cd34+ Cell Mobilization. *Stem Cells Dev* (2008). 10.1089/scd.2008.0027 18393627

[35] Prat M (2006). Use of flt3 ligand to evaluate residual hematopoiesis after heterogeneous irradiation in mice. Radiat Res.

[36] Ganz T (2019). Erythropoietic regulators of iron metabolism. Free radical biology & medicine.

[37] Girelli D, Nemeth E, Swinkels DW (2016). Hepcidin in the diagnosis of iron disorders. Blood.

[38] Arezes J (2018). Erythroferrone inhibits the induction of hepcidin by BMP6. Blood.

[39] Kautz L (2014). Identification of erythroferrone as an erythroid regulator of iron metabolism. Nature genetics.

[40] Ganz T (2011). Hepcidin and iron regulation, 10 years later. Blood.

[41] Wu G (1998). Intestinal Mucosal Amino Acid Catabolism. The Journal of nutrition.

[42] Wu G (2009). Amino acids: metabolism, functions, and nutrition. Amino Acids.

[43] Bannister L (2016). Environmentally Relevant Chronic Low-Dose Tritium and Gamma Exposures do not Increase Somatic Intrachromosomal Recombination in pKZ1 Mouse Spleen. Radiat Res.

[44] Priest ND (2017). Tritium (3 H) Retention In Mice: Administered As HTO, DTO or as 3 H-Labeled Amino-Acids. Health physics.

[45] Roch-Lefevre S (2018). Cytogenetic damage analysis in mice chronically exposed to low-dose internal tritium beta-particle radiation. Oncotarget.

[46] Muckenthaler MU, Rivella S, Hentze MW, Galy B (2017). A Red Carpet for Iron Metabolism. Cell.

[47] Hentze MW, Muckenthaler MU, Galy B, Camaschella C (2010). Two to tango: regulation of Mammalian iron metabolism. Cell.

[48] Roe MA, Collings R, Dainty JR, Swinkels DW, Fairweather-Tait SJ (2009). Plasma hepcidin concentrations significantly predict interindividual variation in iron absorption in healthy men. The American journal of clinical nutrition.

[49] Young MF (2009). Serum hepcidin is significantly associated with iron absorption from food and supplemental sources in healthy young women. The American journal of clinical nutrition.

[50] Fanjul C, Barrenetxe J, Lostao MP (2013). Basal leptin regulates amino acid uptake in polarized Caco-2 cells. Journal of physiology and biochemistry.

[51] Shinoda S, Yoshizawa S, Nozaki E, Tadai K, Arita A (2014). Marginally excessive iron loading transiently blocks mucosal iron uptake in iron-deficient rats. American journal of physiology. Gastrointestinal and liver physiology.

[52] Balonov MI, Muksinova KN, Mushkacheva GS (1993). Tritium radiobiological effects in mammals: review of experiments of the last decade in Russia. Health physics.

[53] Flegal M, Blimkie M, Roch-Lefevre S, Gregoire E, Klokov D (2013). The lack of cytotoxic effect and radioadaptive response in splenocytes of mice exposed to low level internal beta-particle irradiation through tritiated drinking water *in vivo*. International journal of molecular sciences.

[54] Pfaffl MW (2001). A new mathematical model for relative quantification in real-time RT-PCR. Nucleic acids research.

